# Prognostic Value of Negative Emotions on the Incidence of Breast Cancer: A Systematic Review and Meta-Analysis of 129,621 Patients with Breast Cancer

**DOI:** 10.3390/cancers14030475

**Published:** 2022-01-18

**Authors:** Cong Xu, Kumar Ganesan, Xiaoyan Liu, Qiaobo Ye, Yuenshan Cheung, Dan Liu, Shaowen Zhong, Jianping Chen

**Affiliations:** 1 School of Chinese Medicine, LKS Faculty of Medicine, The University of Hong Kong, Hong Kong; 62442msw@gmail.com (C.X.); kumarg@hku.hk (K.G.); yuenshan_cheung@yahoo.com.hk (Y.C.); 2 Department of Breast Surgery, Guangdong Provincial Hospital of Traditional Chinese Medicine, Guangzhou 510405, China; 13660796179@163.com (X.L.); 13760791299@163.com (D.L.); 3 School of Basic Medical Sciences, Chengdu University of Traditional Chinese Medicine, Chengdu 610075, China; yeqiaobo@cdutcm.edu.cn; 4 Shenzhen Institute of Research and Innovation, The University of Hong Kong, Shenzhen 518057, China

**Keywords:** breast cancer, negative emotions, emotion types, follow-up, prognosis

## Abstract

**Simple Summary:**

Negative emotions (NEs) and their impacts have greatly influenced the incidence and risk of breast cancer (BC). The present study aims to provide an association between NEs and the incidence of BC with possible risk factors. A total of 9343 studies were screened; nine studies met all inclusion criteria that were considered for the meta-analysis. Observational studies were included with relative risks (RR) and corresponding 95% confidence intervals (CI). We analyzed data from 129,621 women diagnosed with NEs of which 2080 women were diagnosed with BC and their follow-up year ranges were from 4–24 years. NEs were significantly associated with a higher incidence of BC with other high-risk factors including, geographical distribution, emotion types, standard diagnosis of NEs, and follow-up duration. This study suggests that NEs significantly increase the risk for the incidence of BC, which can be supportive of the prognosis of the disease.

**Abstract:**

Breast cancer (BC) is one of the common malignant tumors in women and affects 1.6 million new cases globally each year. Investigators have recently found that negative emotions (NEs) and their impacts have greatly influenced the incidence and risk of BC. The present study aims to provide an association between NEs and the incidence of BC with possible risk factors. A total of 9343 studies were screened; nine studies met all inclusion criteria that were considered for the meta-analysis. The qualitative studies were measured by the Newcastle-Ottawa Scale; the observational studies were included with relative risks (RR) and corresponding 95% confidence intervals (CI). Besides the NEs and BC, the possible risk factors were evaluated. We analyzed data from 129,621 women diagnosed with NEs of which 2080 women were diagnosed with BC and their follow-up year ranges were from 4–24 years. NEs were significantly (*p* < 0.0001) associated with a higher incidence of BC with RR = 1.59, 95% CI:1.15–2.19, with other high-risk factors including, geographical distribution, emotion types, standard diagnosis of NEs, and follow-up duration. This study suggests that NEs significantly increase the risk for the incidence of BC, which can be supportive of the prognosis of the disease.

## 1. Introduction

Breast cancer (BC) is the second foremost cause of cancer demise in women globally. According to the Global Cancer Statistics 2020, the newly affected cases of BC have surpassed lung cancer to become the first leading cancer in the world [1]. BC has become one of the main threats to females due to the high incidence rate [1,2] and mortality rate [3]. Among women, BC accounts for 1 in 4 cancer cases and 1 in 6 cancer deaths, ranking first for incidence in the majority of nations (159 of 185 nations) and for the high death rate in 110 nations [4]. The increased incidence rates of BC could be based on the hormonal, reproductive risk factors (early menarche, advanced maternal age pregnancy, late-onset menopause, less number of children, less breastfeeding, use of oral contraceptives and hormonal therapy), negative emotions (NEs, depression, anxiety, psychosis, and psychological factors) and lifestyle (obesity, alcohol intake, smoking, lacking physical activity) [5,6,7,8]. Recently, researchers found that NEs can affect human endocrine and immune function, which in turn can affect the incidence and development of BC [9]. BC patients receive an extensive period of multimodal treatment, including surgery, radiotherapy, chemotherapy, and invasive treatment, which are frequently accompanied by changes in physical status and function, unpleasant side effects, and declining quality of life [10,11]. Thus, BC patients continuously suffer from NEs under chronic psychological stress. Apart from treatment-related distress, the cancer diagnosis can also aggravate NEs.

Several clinical studies have also suggested that patients with BC suffer a higher incidence of mental disorders compared with the general population [7,12,13]. Anxiety and depression are the most common NEs experienced by most BC patients and the incidence rates are very high during the BC treatment, which was 70% and 60%, respectively [14,15]. These NEs may worsen the treatment, lead to greater pain, a longer stay in the hospital, increase the risk of disease progression, and affect the quality of life, and death [16]. Emotions are multifaceted and widespread human experiences, which occur in two phases namely positive (happiness) or negative (sadness), that can occur rapidly and mechanically. According to the Positive and Negative Affect Schedule (PANAS) scale, the positive affect (PA) represents the enthusiastic, active, and alert, which is a state of high energy, full concentration, and pleasurable engagement. The negative affect (NA) is a general dimension of subjective distress and unpleasable engagement that subsumes a variety of aversive mood states, including anger, contempt, disgust, guilt, fear, and nervousness. Low PA and high NA are the distinguished features of depression and anxiety that generate altered behavioral, physiological, and subjective responses [17]. Patients with BC generally suffer from significant clinical depression even years after diagnosis and treatment [18], which impair physiological and psychological function, and can lead to other serious complications including severe illnesses and even death [19,20].

Earlier studies suggested that several factors connect to depressive symptoms in BC patients, including demographics, disease-related factors, and distinct psychosocial characteristics such as coping styles and personality traits [21,22,23,24]. Emotional suppression is one of the coping style strategies, whereby, the individual can intentionally regulate the expression of NEs including, anxiety, anger, and sadness. Earlier clinical data have also confirmed the association between emotional suppression and psychosocial maladjustment such as depressive symptoms in patients with BC [25,26,27]. For instance, Li et al. [28] found that anger suppression was connected with the advanced level of depression in BC individuals during chemotherapy. Similarly, Kugbey et al. [12] found that emotional suppression was highly connected to the worsening mood in women with advanced BC. All these findings suggest that anger or emotional suppression worsen the mood in women with BC, which promotes a high incidence of BC progression.

Depression or anxiety can influence the physical and psychological function and the quality of patients with BC. The type and severity of life events and the accumulation of the number of life events can increase the psychological burden with NEs that increases the risk of BC [29,30]. Previously, a five year observational and cohort study was conducted in 222 women with early BC by an NHS breast clinic, London and the outcome revealed that around 50% of the women with early BC had depression, anxiety, or both in the year after diagnosis [21]. Lueboonthavatchai [31] conducted a study in Thailand and found that anxiety and depression are common disorders in BC, which were caused due to psychosocial factors. In addition, social and other personal factors also influence the high incidence of BC [32]. The animal model has also proved that psychological stress can promote tumor growth [33]. Contrarily, some studies show that NEs do not have any role in increasing BC risk [34,35,36]. Preceding meta-analyses confirmed that NEs were related to mortality [20,37] but not to progression in cancer patients [20]. Moreover, these studies were constrained by high heterogeneity based on the types of cancers. However, BC is a hormone-dependent malignancy, and its response to psychological disorders could be distinct from other malignancies. Hereafter, it is required to determine the relationship between NEs and the BC. Thus, the present study aims to provide the association between NEs and BC and their possible risk factors, which can be beneficial for the prognosis of BC progression.

## 2. Materials and Methods

### 2.1. Protocol and Registration

We followed the standard preferred reporting items for systematic reviews and meta-analysis (PRISMA) criteria [38] when conducting this meta-analysis and reporting the results. A predetermined review protocol was registered (CRD42021262126) in the PROSPERO database.

### 2.2. Search Strategy

Following recommendations of the meta-analysis of observational studies in Epidemiology [39], we conducted a systematic literature search for articles on NEs and BC, which were published until 30 June 2021 using the following electronic databases of PubMed, Embase, and the Cochrane Library. The search strategy was implemented using combined index terms (medical subject headings, Emtree) and free-text keywords. The keywords included (“breast cancer” OR “breast carcinoma”) AND (“depression” OR “psychological factor” OR “stress” OR “anxiety” OR “psychosis”) AND (“cohort study” OR “follow-up study” OR “case-control study). There were a total of 9343 results from three databases; 143 of them were duplicated. In addition, the reference lists retrieved articles that were manually scrutinized to detect potentially relevant studies.

### 2.3. Inclusion and Exclusion Criteria

**Figure 1:**
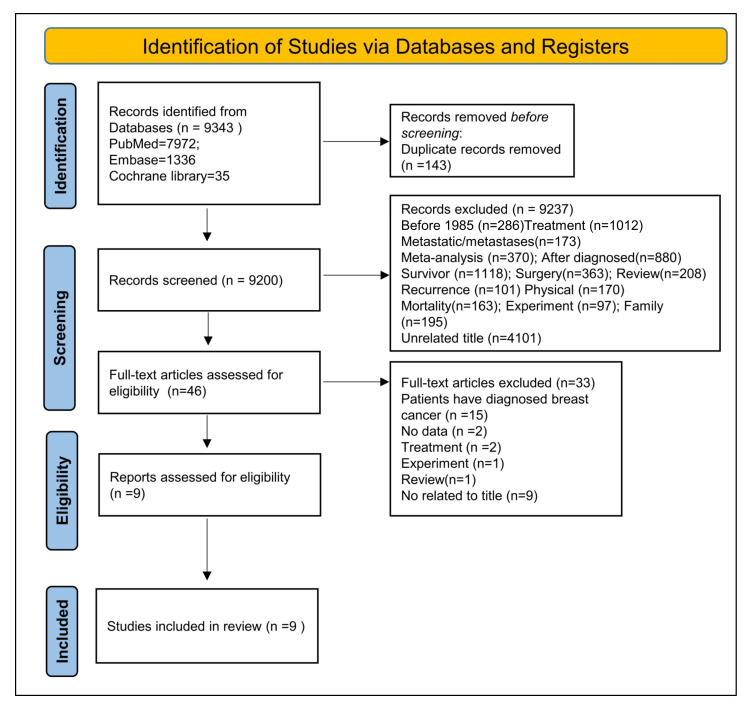
Selection of studies for inclusion in the meta-analysis based on PRISMA guidelines.

Two of our team members independently evaluated the studies for inclusion, and studies were included in the meta-analysis if they met the following criteria: (1) the type of the designed study was cohort (prospective, follow-up, or longitudinal studies) or case-controlled studies; (2) NEs assessment met recognized domestic and foreign standards; (3) BC assessment met clinical diagnostic criteria; (4) the relative risk of statistical indicators was fully reported in the article (relative risk, RR) or ratio (odds ratio, OR) and its 95% confidence interval (CI); (5) the main content of the literature was the relationship between NEs and the risk of BC. In addition, multiple groups of data were also included. The studies were excluded if they (1) were the type of literature: review, conference, review, lectures, or abstract articles; (2) did not have data, incomplete, or low-quality studies; (3) were animal experiments or basic research; (4) were younger than 17 years old; (5) included male patients only; (6) did not mention any possible risk factors in the paper; (7) were not in English. We retrieved primary studies of individuals’ BC risk models observing each database from its inception up to 30 June 2021. After complete analysis, 9334 studies were eliminated; nine studies were left for further analysis. Details of the searching and study selection process are listed in Figure 1.

### 2.4. Data Extraction and Quality Assessment

Two reviewers independently extracted the data from the included studies. In case of disagreement between researchers, the inclusion of studies was determined by consensus. We reported the outcome of this process with a PRISMA flowchart. The following details were presented in this review: author, age, year of publication, country, sample size and characteristics, follow-up duration, type and measurement of NEs (depression, anxiety, psychosis), and the case of the BC. The quality of the studies was assessed using the Newcastle-Ottawa Quality Assessment Scale (NOS) [40] (Table 1). The scale was ranked as follows: poor quality (0 or 1 star in selection domain OR 0 stars in comparability domain OR 0 or 1 stars in outcome/exposure domain), fair quality (2 stars in selection domain AND 1 or 2 stars in comparability domain AND 2 or 3 stars in outcome/exposure domain), and high quality (3 or 4 stars in selection domain AND 1 or 2 stars in comparability domain AND 2 or 3 stars in outcome/exposure domain). Even though this study focused on the NEs, BC risk, and the related risk factors, it was necessary to analyze the incidence rate. Furthermore, the follow-up study was also noteworthy. The score of the outcome column was low, however, the quality of the examined paper was not affected. One of the reviewers extracted the data for analysis, which include: publication year, source of the data, sample size, study design, BC, and possible risk factors, viz., country, type of emotions, standard diagnosis, follow-up years (Table 2).

### 2.5. Statistical Analysis

This meta-analysis of the included studies was performed by Review Manager 5.4 software on cochrane.org. (accessed on 15 August 2021). The exact binomial 95% CI of RR in each study was achieved. The HR was replaced with the RR if the RR was not reported. For dichotomous outcomes, data were separated into groups for analysis, the pooled RR with 95% CI was calculated using Review Manager 5.4 with a random-effect model. The I^2^ statistic was used to evaluate heterogeneity of all the studies, and if the value of I^2^ > 50%, meant the study was considered statistically significant [41] and we used the random-effect model [42]. The funnel plots were used to detect the risk of bias. All nine eligible studies were included in this analysis.

## 3. Results

### 3.1. Study Selection and Characteristics

After identifying 9343 references, 143 duplicate publications and 9237 irrelevant studies were excluded, leaving 46 potentially eligible studies. Finally, nine cohort studies were included in the meta-analysis (Figure 1). The basic information of included studies is listed in Table 1. The duration of follow-ups ranged from 4–24 years. A total of 129,621 BC patients with NEs were involved; among the nine studies, four studies were from the USA, two were from Europe, three were from Asia, and one was from Israel. Mental symptoms were assessed by several tools such as the Center for Epidemiological Studies-Depression (CES-D), Beck Depression Inventory (BDI), General Health Questionnaire-12 (GHQ-12), while clinical mental disorders were diagnosed based on the International Classification of Diseases (ICD), Diagnostic and Statistical Manual of Mental Disorders (DSM-MD), Brief Symptom Inventory (BSI), Perceived Stress Scale (PSS) or questionnaire (Table 3).

**Table 1:** Newcastle-Ottawa Quality Assessment Scale of the study.

Authors	Selection				Comparability	Outcome			
	Representativeness of the Exposed Cohort	Selection of the Non-Exposed Cohort	Ascertainment of Exposure	Demonstration that Outcome of Interest Was Not Present at the Start of the Study	Comparability of Cohorts Based on the Design or Analysis Controlled for Confounders	Assessment of the Outcome	Was Follow-Up Long Enough for Outcomes to Occur	Adequacy of the Follow-Up of Cohorts	Total Score
Aro et al., 2005 [43]	0	0	1	1	1	0	1	1	5
Mitchell et al. 2017 [44]	1	1	1	1	1	1	1	1	8
Chang et al., 2015 [45]	1	1	1	1	2	1	1	1	9
Jacobs and Bovasso, 2000 [46]	1	0	1	1	1	1	1	1	7
Hjerl et al., 1999 [47]	1	1	1	1	2	1	1	1	9
Wakai et al., 2007 [48]	1	0	1	1	1	1	1	1	7
Yeh and Lee, 2016 (Anxiety) [49]	1	1	1	1	2	1	1	1	9
Yeh and Lee, 2016 (Depression) [49]	SAA	SAA	SAA	SAA	SAA	SAA	SAA	SAA	SAA
Hahn and Petitti, 1988 [50]	1	1	1	1	1	1	1	1	8
Peled et al., 2008 [51]	1	1	1	1	2	1	1	1	9

**Table 2:** Information of included studies.

Authors	Year	Country	RR Ratio	Sample Size	BC Cases	Follow-Up Years	Age	Emotion Type	Diagnosis of NEs
Aro et al., 2005 [43]	2005	Finland	2.83 (1.26–6.36)	10,893	278	6–9	48–50	Psychological risk factors	BDI
Mitchell et al., 2017 [44]	2017	USA	1.36 (0.31–5.94)	1067	43	24	19–79	Depression	DSM-MD III
Chang et al., 2015 [45]	2015	USA	3.8 (1.0–14.2)	3109	25	13	>18	Depression	DSM-MD III
Jacobs and Bovasso, 2000 [46]	2000	USA	17.2 (3.76–77.08)	1533	40	15	40	Stress	MDD
Hjerl et al., 1999 [47]	1999	Denmark	1.02 (0.97–1.08)	66,648	1270	24	>15	Affective orneurotic disorders	DSM-MD III
Wakai et al., 2007 [48]	2007	Japan	1.01 (0.69–1.5)	34,497	34	7.5	40–79	Stress	Questionnaire
Yeh and Lee, 2016 (Anxiety) [49]	2016	Taiwan	2.173 (1.009–4.648)	1160	10	5.19	17–70	Anxiety	PSS
Yeh and Lee, 2016 (Depression) [49]	2016	Taiwan	4.979 (1.643–12.303)	1160	5	5.19	17–70	Depression	PSS
Hahn and Petitti, 1988 [50]	1988	USA	1.5 (0.9–2.5)	8932	120	18	-	Depression	ICD-8
Peled et al., 2008 [51]	2008	Israel	1.62 (1.09–2.4)	622	255	4	<45	Psychological distress	BSI
Total				129,621	2080				

Mental symptoms were assessed by several tools: The International Classification of Diseases (ICD), Diagnostic and Statistical Manual of Mental Disorders III (DSM-MD III); Perceived Stress Scale (PSS); Brief Symptom Inventory (BSI); Major depressive disorder (*MDD*); Beck Depression Inventory (BDI).

**Table 3:** The characteristics of the subgroup.

Authors	Country			Type of Emotion			Follow-Up Years			Outcome		Diagnosis		
	USA	Europe	Asia	Depression	Psychosis	Psychological Factor	<10	10–20	>20	Positive	Negative	ICD-8	DSM-III	Others
Aro et al., 2005 [43]		√				√	√				√	√		
Mitchell et al., 2017 [44]	√			√					√	√			√	
Chang et al., 2015 [45]			√	√				√			√		√	
Jacobs and Bovasso, 2000 [46]	√					√		√			√	√		
Hjerl et al., 1999 [47]		√			√				√		√	√		
Wakai et al., 2007 [48]			√			√	√				√	√		
Yeh and Lee, 2016 (Anxiety) [49]			√		√				√	√		√		
Yeh and Lee, 2016 (Depression) [49]			√	√					√		√	√		
Hahn and Petitti, 1988 [50]	√			√				√			√			√
Peled et al., 2008 [51]			√	√			√			√				√

### 3.2. Effect of the NEs on the Incidence of BC

**Figure 2:**
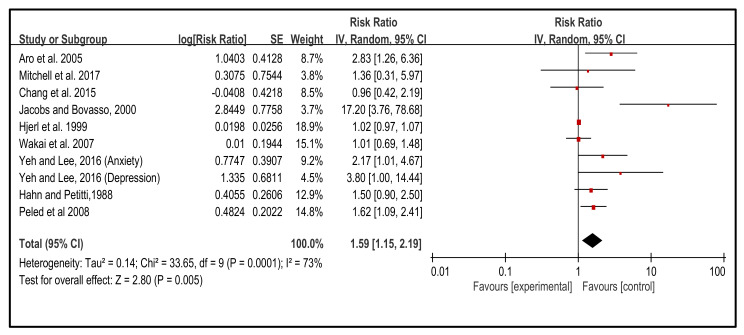
Effect of the NEs on the incidence of BC [43,44,45,46,47,48,49,50,51].

The outcome of the meta-analysis showed that the influence of NEs on the incidence of BC was significant (*p* < 0.005) (RR, 1.59, 95% CI (1.15, 2.19) (Figure 2). The heterogeneity of this analysis was relatively high (I^2^ = 73%), as included studies covered the data across countries, and all individual variations had affected the result. Moreover, distinct inclusion criteria of individual studies also affected the heterogeneity. Therefore, subgroup analysis was required to observe the potential risk factors and their influence on BC. Our outcome showed the RR of the incidence of BC has been associated with all the subgroup analyses. Based on the findings, it was clear the influence of NEs on the incidence of BC.

### 3.3. Effect of Emotion Types on the Incidence of BC

**Figure 3:**
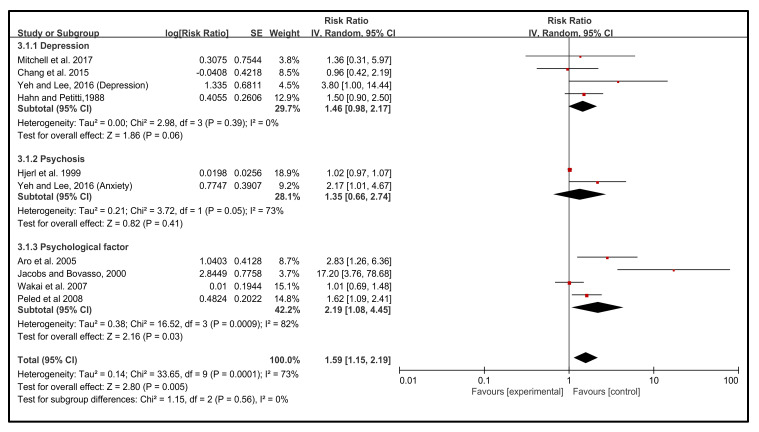
Emotion type. The results were stratified by different emotion types – depression, psychosis, and psychological factors [43,44,45,46,47,48,49,50,51].

Emotion types are one of the contributing risk factors for the incidence of BC. NEs are classified and analyzed according to psychological factors, psychosis, and depression, which significantly influenced the incidence of BC. Psychological factors contributed 42.2% more (RR 2.19, 95% CI (1.08, 4.45), depression 29.7% more (RR 1.46, 95% CI 0.98, 2.17) and psychosis 28.1% more (RR 1.35, 95% CI 0.66, 2.74) likely for the incidence of BC. The heterogeneity of this analysis was relatively high (I^2^ = 73%). The psychological factor significantly contributed (42.2% more) to the incidence of BC than the depression (29.7%) and psychosis (28.1%) examined; the overall effect was *p* = 0.005 (Figure 3).

### 3.4. Effect of Geographical Distribution on the Incidence of BC

**Figure 4:**
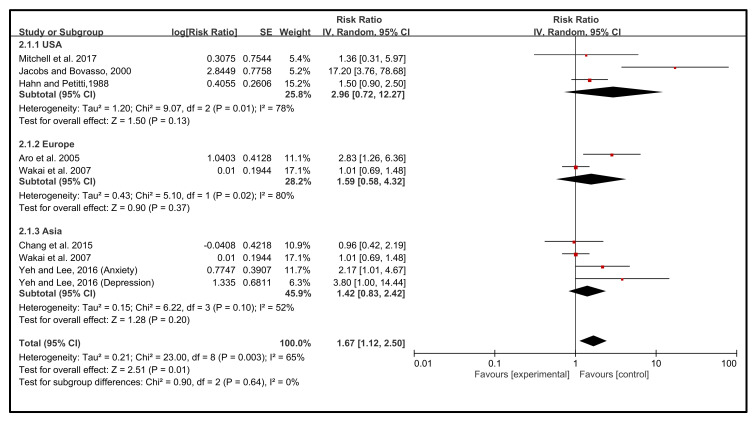
Geographical distribution. The results were stratified by different nations [43,44,45,46,48,49,50].

Geographical distribution was found to be a contributing risk factor for the incidence of BC as the NEs in regard to BC differ according to nation. NEs were considerably more common in the Asian women (45.9%) (RR 1.42, 95% CI 0.83, 2.42), followed by 25.8% in USA (RR 2.96, 95% CI 0.72, 12.27) and 28.2% in Europe (RR 1.59, 95% CI 0.58, 4.32). The heterogeneity of this analysis was relatively higher (I^2^ = 65%) and the overall effect was significant (*p* = 0.01). These higher levels of heterogeneity were due to the rough division of the population, different races, and ethnicity that may lead to the bias (Figure 4). 

### 3.5. Effect of Follow-Up Years on the Incidence of BC

**Figure 5:**
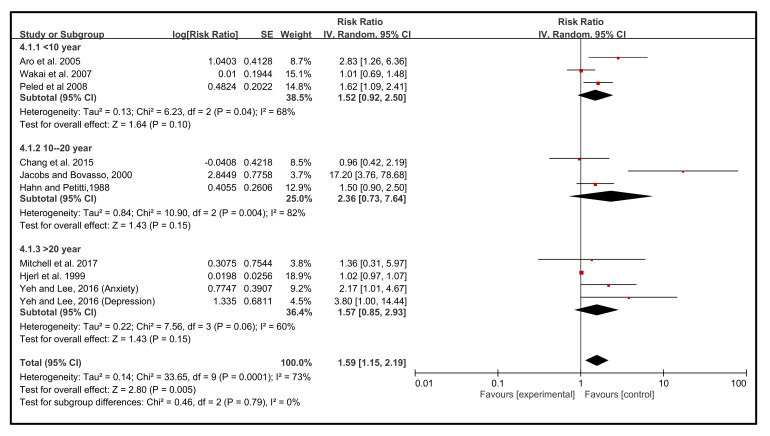
Follow-up years. The results were stratified by follow-up duration [43,44,45,46,47,48,49,50,51].

The follow-up years are also found to be a significant (*p* = 0.005) contributing risk factor for the incidence of BC. NEs occurred considerably in < 10 years (38.5%) (RR 1.52, 95% CI 0.92, 2.50), followed by 36.4% in >20 years (RR 1.57 95% CI 0.85, 2.93) and 25% in 10–20 years (RR 2.36, 95% CI 0.73, 7.64). The heterogeneity of this analysis was relatively moderate (I^2^ = 60%) and the test for subgroup differences was about 0%, and RR ratios were nearly identical, ranging from 1.15 to 2.19 (Figure 5).

### 3.6. Effect of Diagnosis of the NEs on the Incidence of BC

**Figure 6:**
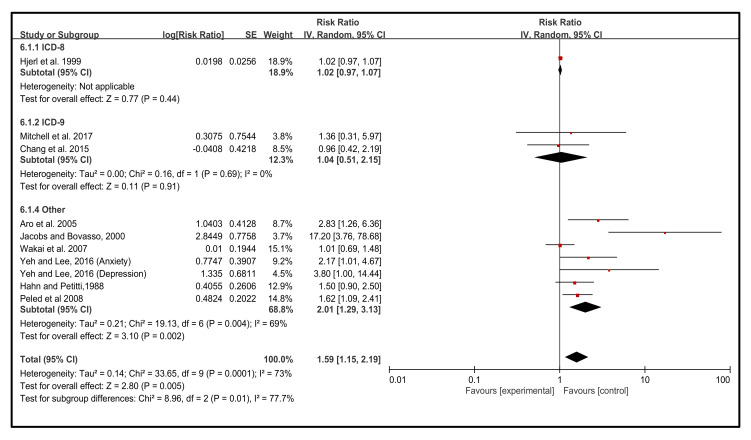
Diagnosis of the NEs. The results were stratified by the diagnosis of the NEs [43,44,45,46,47,48,49,50,51].

The standard diagnosis is another significant (*p* = 0.005) contributing risk factor for the incidence of BC. NEs were considerably diagnosed by ICD-8 (18.9%) (RR 1.02, 95% CI 0.97, 1.07), followed by 12.3% ICD-9 (RR 1.04 95% CI 0.51, 2.15). However, other standard methods were significantly (*p* = 0.002) used for the diagnosis of NEs, which accounted for 68.8% (RR 2.01, 95% CI 1.29, 3.13). The heterogeneity of this analysis was relatively high (I^2^ = 73%) and the test for subgroup differences was about 77%, and RR ratios were nearly identical, ranging from 1.15 to 2.19 (Figure 6).

### 3.7. Sensitivity Analysis and Publication Bias

**Figure 7:**
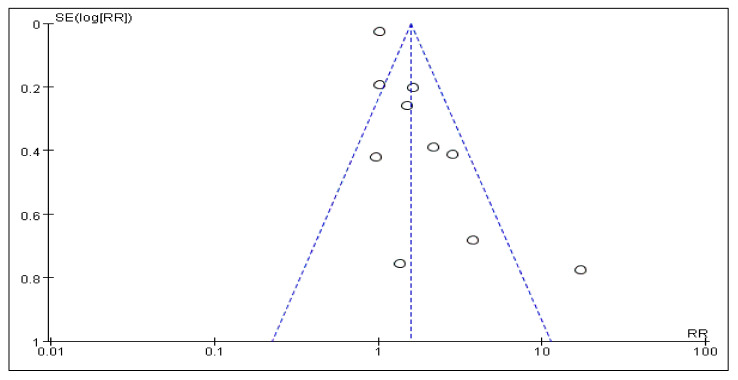
Funnel plot for publication bias analysis.

Due to diverse factors such as emotion types, geographical distribution, follow-up years, standard diagnosis, etc., there was no evidence of publication bias based on visual inspection of funnel plots. Figure 7 showed the Beggs funnel plot of tests related to NEs on the incidence of BC. Sensitivity analysis was performed to assess the stability of the results, and a single study was omitted each time from the meta-analysis. The results showed that the corresponding pooled estimates were not significantly altered, indicating that no individual study influenced the results. Interpretation of this plot showed no signs of publication bias in these studies.

## 4. Discussion

Various clinical investigations have proposed that patients with BC suffer a much higher incidence of NEs when compared to the general population [7,12,13]. These NEs may worsen the diagnosis and treatment, lead to an elevated risk of disease progression, and affect the quality of life, and death [16]. Anxiety and depression are the most common NEs experienced by most BC patients. The present study validates that depression, anxiety, and other psychological factors are greatly connected to the increase in the incidence of BC. In our study, depression was predicted to incur a 30% approximate increase in the incidence rate of BC risk among cancer patients, which was more severe than the increased risk of 18% as analyzed by Pinquart et al. [37], or 25% by Satin et al. [20], but is consistent with the 30% presented by Wang et al. [7]. This outcome postulates an increased tendency in the highest mortality risk nowadays. Notably, in contrast to preceding depression-focused meta-analyses, our present outcomes also confirm that psychological factors can envisage higher incidence rates (42.2%) in patients with BC. The incidence rates of BC are also higher in patients with depression (29.7%) or psychosis (28.1%). Psychosis and comorbid depression can also increase the risk of higher incidence rates and cancer-specific mortality [52]. Earlier meta-analysis showed that psychosis was associated with a significantly increased risk of BC incidence in women with significant heterogeneity (*p* < 0.001; I^2^ = 89%) [52]. Our results also suggest that psychosis and depression are stronger risk factors for BC incidence. Bearing in mind that psychosis and depression co-occur clinically, more care should be given to them during the treatment of BC patients.

In addition to our present study, anxiety and other mental illness such as stress and dysthymic also could affect the incidence of BC [7,15,23,53]. Considering that NEs, combining anxiety and depression may appear in the BC patient at the same time, which may greatly increase the risk of BC. Hence, more attention has to be paid during the treatment and screening of the NE individuals. Controversially, in a national representative health survey study from Finland, Knekt et al. found that there is no compulsory connection between depression and the incidence of BC [54]. A prospective cohort study conducted on the elderly population found that long-term depression can elevate the risk of BC [55]. Several factors may affect the incidence of BC including lifestyle, significant events that occurred suddenly in day-to-day life, character, behavioral factors, and biological factors [56]. In recent studies, many life events can contribute to the NEs such as unemployment or material status (single or divorced), which may influence the incidence of BC [32,51].

In a study conducted on 1400 Chinese women with BC, those on a low-income were likely to have greater depression than the high-income women [32]. In another cohort study, divorced or single women (16.4%) and widowed (18.9%) were prone to a higher incidence of BC when compared to married women [37,57]. Another noteworthy subject was the relationship between daily stress and the incidence of BC. In a 24-year cohort study, the effect of daily stress on the risk of BC was observed. Based on the outcomes, the women with daily stress were twice as likely to be at risk of BC than the women who had no or minimal mental illness [38]. In a study conducted on 622 young women, when two or more events happened to the young women, the odds ratio of the BC incidence OR = 1.62, 95% CI (1.09, 2.40); the outcomes showed that experiencing more negative life events can increase the risk of BC [51]. NEs comprise of depression, anxiety, and psychological disorders that are highly connected to various biological mechanisms, including the activation of the hypothalamic-pituitary-adrenal axis in patients [58,59]. Alteration in the levels of the female hormone can influence the incidence of BC. According to WHO randomized trial findings, high expression of estrogen plus progesterone is associated with the increased incidence of BC [60]. Recent study findings suggested that patients with depression and severe mental illness, who had received hormonal therapy, have a high incidence of BC with 48.8% and 43%, respectively [61].

This study has various implications for clinical practice. It is key to raising awareness amongst healthcare professionals acting at different levels of the healthcare system of the elevated risk of mental health symptoms among BC, specifically psychosis, depression, and other psychological factors. Screening for these mental health disorders in BC patients can merit further investigation. Predictors of distress among BC individuals include having perceived functional limitations, psychiatric history, menopausal stage, fatigue, lower socioeconomic status, and modifiable factors such as vasomotor symptoms, pain, less social support, physical activity, and smoking [53]. Psychosocial care and regular monitoring of patient-reported outcomes during treatment care are possible to reduce the burden of these environments [62].

Since the value of NEs on BC differs nationwide, geographical distribution is also a noteworthy contributing risk factor for the incidence of BC. NEs were considerably more common in Asian women (45.9%), followed by the USA (25.8%) and Europe (28.2%). Our study was inconsistent with earlier reports between 1988–92 regarding the incidence of BC being the highest among women in the USA, followed by Africans, Asians, Europeans, and Hispanics [63,64]. Depression was the highest and strongest gradient effect in most BC patients in the general inpatient population; thus increasing numbers of comorbidities, including mental health-related, negatively impact BC prognosis, and increase BC mortality [65].

Earlier systematically reviewed investigations provided the data for NEs in BC individuals (>2 years follow-up) and healthy individuals. The outcomes indicated that NEs worsen the incidence of BC and increase the stages among BC patients [66]. Maass et al. [67] also demonstrated a higher frequency of NEs symptoms occurring among BC patients (>1-year follow-up). Our present study shows that NEs occurred considerably in <10 years (38.5%), >20 years (36.4%), and 10–20 years (25%). Our study results are consistent with earlier other systematic reviews on the topic assessed by the different follow-up years and the incidence of psychological factors and depressive symptoms in patients with BC [67,68,69,70,71]. The standard diagnosis is a noteworthy contributing risk factor for the incidence of BC. NEs were considerably diagnosed by ICD-8 (18.9%), ICD-9 (12.3%), and other standard methods (68.8%). Our study outcomes are consistent with earlier other systematic reviews on the topic assessed by the different standard diagnoses and the incidence of BC [7,72,73]. Previously, NEs suppression had a substantial effect on depressive and psychosis symptoms, which were also significantly correlated with CES-D scores [28]. This outcome was parallel to the findings of Iwamitsu’s study [74], which implied that suppression of different NEs had different psychological outcomes. Thus, it is essential to encourage BC patients to express their NEs correctly and appropriately.

### Implications

This study has various implications for clinical practice. It is key to raising awareness amongst healthcare professionals acting at different levels of the healthcare system of the elevated risk of mental health symptoms among BC, specifically psychosis, depression, and other psychological factors. Screening for these mental health disorders in BC patients can merit further investigation. Predictors of distress among BC individuals include having perceived functional limitations, psychiatric history, menopausal stage, fatigue, lower socioeconomic status, and modifiable factors such as vasomotor symptoms, pain, less social support, physical activity, and smoking [53]. Routine screening and early detection are highly recommended. As NEs may alter across the trajectory of cancer care, screening at consistent intervals can effectively aid in monitoring psychological alterations in the early period and permit for timely intervention to avert it from worsening. Also, our study directly emphasizes the implication of clinical therapy for NEs in a patient with BC. It is alleged that psychotherapy and mind–body treatments may release NEs symptoms in BC patients [7]. Psychosocial care and regular monitoring of patient-reported outcomes during treatment care are possible to reduce the burden of these situations [62].

## 5. Conclusions

Based on the present findings, the study suggests that NEs have adverse effects and prognostic tools for the incidence of BC. The results directly support the requirement for early and periodical detection and timely treatment of mental disorders in patients. Furthermore, physicians, psychiatrists, and oncologists should organize and develop increased options in the coordination of care and treatment for patients with BC. Regular physical exercise, yoga, indoor and outdoor activities, promoting a positive body image, and self-esteem improvement are key points to improve the NEs in BC patients. Early intervention in mental health can be reinforced to reduce the mental health disease as well as the incidence of BC, which may release NEs symptoms in BC patients.
